# Supplementary material for: Evaluating Two Educational Interventions for Enhancing COVID-19 Knowledge and Attitudes in a Sample American Indian/Alaska Native Population
Source: Vaccines (Basel). 2024 Jul 17;12(7):787. doi: 10.3390/vaccines12070787 (PMC11281502; doi:10.3390/vaccines12070787)
Supplement: Supplementary file 1 [file vaccines-12-00787-s001.zip › Supplementary Material S1.pdf]

# Pre-survey, Educational Intervention, Post-survey

Start of Block: Pre-survey

*

SURVEY_ID Please enter the last three digits of your **zip code** and the last three digits of your **phone number.**
This will be your unique 6-digit identifier number. Your identity will remain anonymous.

________________________________________________________________

Page Break

DEMO **Demographic Information**

DEMO1 How would you describe your gender?

- ○ Female (1)
- ○ Male (2)
- ○ Transgender Female (3)
- ○ Transgender Male (4)
- ○ Gender Variant/Non-Conforming (5)
- ○ Other (please specify): (6) ________________________________________________
- ○ Prefer Not to Answer (7)

DEMO2 What is your age?

- ○ 18 – 24 years old (1)
- ○ 25 – 34 years old (2)
- ○ 35 – 44 years old (3)
- ○ 45 – 54 years old (4)
- ○ 55 – 64 years old (5)
- ○ 65+ years old (6)

DEMO4 Please specify your ethnicity.

- ◯ Hispanic (1)
- ◯ Non-Hispanic (2)

---

DEMO5a Current Residence: City/Town Name

________________________________________________________________

---

DEMO5b Current Residence: Zip Code

________________________________________________________________

---

DEMO 8 Do you usually think of yourself as a Republican, a Democrat, an Independent, or something else?

- ◯ Republican (1)
- ◯ Democrat (2)
- ◯ Independent (3)
- ◯ Something else (4)

---

Page Break

DEMO10 Was <u>your employment status</u> impacted by the COVID-19 pandemic?

- ○ Yes (1)
- ○ No (0)
- ○ Not applicable (2)

---

DEMO11 Are you or any person in your household considered an <u>essential worker</u>?
An *essential worker* is expected to go to work despite a stay-at-home order.
***Select <u>all</u> that apply.***

- ▢ I am an essential worker. (1)
- ▢ Someone else in my household is an essential worker. (2)
- ▢ No one in my household is an essential worker. (4)

---

DEMO12
Since January 2020, have you been <u>tested</u> for the COVID-19 virus?

- ○ Yes (2)
- ○ No (0)
- ○ Not sure (1)

---

DEMO13 Have you or anyone in your social circle (family, friends, acquaintances, etc.) ever tested positive for COVID-19?

- ◯ Yes (2)
- ◯ No (0)
- ◯ Don't know (1)

---

DEMO14 Do you personally know someone who has been hospitalized or died from COVID-19?

- ◯ Yes (2)
- ◯ No (0)
- ◯ Don’t know (1)

---

DEMO15 Have <u>you</u> ever been <u>diagnosed</u> with any of the following medical conditions?
***Select <u>all</u> that apply.***

- ☐ Cancer (1)
- ☐ Chronic kidney disease (2)
- ☐ Chronic lung diseases (i.e. chronic obstructive pulmonary disease, moderate-to-severe asthma, interstitial lung disease, cystic fibrosis, pulmonary hypertension) (3)
- ☐ Dementia or other neurological conditions (4)
- ☐ Diabetes (type 1 or type 2) (5)
- ☐ Down syndrome (6)
- ☐ Heart conditions (i.e. heart failure, coronary artery disease, cardiomyopathies, or hypertension) (7)
- ☐ HIV infection (8)
- ☐ Immunocompromised state (weakened immune system) (9)
- ☐ Liver disease (10)
- ☐ Overweight and obesity (11)
- ☐ Pregnancy (12)
- ☐ Sickle cell disease or thalassemia (13)
- ☐ Solid organ or blood stem cell transplant (14)
- ☐ Stroke or cerebrovascular disease, which affects blood flow to the brain (15)
- ☐ Substance use disorders (16)

☐ Don’t know (17)

DEMO16 Has anyone in your household (besides you) ever been diagnosed with any of the following medical conditions?
***Select all that apply.***

- ▢ Cancer (1)
- ▢ Chronic kidney disease (2)
- ▢ Chronic lung diseases (i.e. chronic obstructive pulmonary disease, moderate-to-severe asthma, interstitial lung disease, cystic fibrosis, pulmonary hypertension) (3)
- ▢ Dementia or other neurological conditions (4)
- ▢ Diabetes (type 1 or type 2) (5)
- ▢ Down syndrome (6)
- ▢ Heart conditions (i.e. heart failure, coronary artery disease, cardiomyopathies, or hypertension) (7)
- ▢ HIV infection (8)
- ▢ Immunocompromised state (weakened immune system) (9)
- ▢ Liver disease (10)
- ▢ Overweight and obesity (11)
- ▢ Pregnancy (12)
- ▢ Sickle cell disease or thalassemia (13)
- ▢ Solid organ or blood stem cell transplant (14)
- ▢ Stroke or cerebrovascular disease, which affects blood flow to the brain (15)
- ▢ Substance use disorders (16)

☐ Don’t know (17)

DEMO17 Which of the following CDC recommended COVID-19 precautions do you currently follow? ***Select <u>all</u> that apply.***

☐ Wearing a mask (nose and mouth covered) (1)

☐ Limited in-person interactions (2)

☐ Social distancing (6 ft away from others) (3)

☐ Avoiding crowds (4)

☐ Avoiding poorly ventilated spaces (5)

☐ Washing your hands often (6)

☐ Not touching your eyes, nose, and mouth with unwashed hands (7)

☐ Covering coughs and sneezes with a tissue or the inside of your elbow (8)

☐ Cleaning and disinfecting surfaces (9)

☐ Monitoring your own health (i.e. being alert of your own symptoms, taking your temperature) (10)

☐ Additional (please specify): (11) ________________________________________________

Page Break

COVIDsource **The following questions ask about how often you get COVID-19 updates and your primary sources.**

---

COVIDsource1 I get COVID-19 news and updates primarily from the following sources: ***Select <u>all</u> that apply***.

- ☐ Television. If yes, specify the channel(s): (1) ________________________________________________
- ☐ Radio. If yes, specify the station(s): (2) ________________________________________________
- ☐ Print (Newspaper, magazine, etc.). If yes, specify the print(s): (3) ________________________________________________
- ☐ Social media (Facebook, Instagram, Twitter, etc.). If yes, specify the platform(s): (4) ________________________________________________
- ☐ Websites. If yes, specify the website(s): (5) ________________________________________________
- ☐ Healthcare professionals and officials (i.e. doctor, nurse, paramedic, etc.). If yes, specify the professional/official title(s): (6) ________________________________________________
- ☐ Personal relationships (Friends, family, co-workers, etc.). If yes, specify the relationship(s): (7) ________________________________________________
- ☐ Religious leader (Pastor, clergy, minister, etc.). If yes, specify the relationship(s): (8) ________________________________________________
- ☐ Governor of your state. (9)

---

*Carry Forward Selected Choices from "I get COVID-19 news and updates primarily from the following sources: Select all that apply."*

X→

COVIDsource2
How **reliable** do you find the source(s) selected on the previous page to be?
Please skip this page if you did not select any source(s) on the previous page.

| | Not at all (1) | A little (2) | Somewhat (3) | A lot (4) | Completely (5) |
| --- | --- | --- | --- | --- | --- |
| Television. If yes, specify the channel(s): (x1) | ○ | ○ | ○ | ○ | ○ |
| Radio. If yes, specify the station(s): (x2) | ○ | ○ | ○ | ○ | ○ |
| Print (Newspaper, magazine, etc.). If yes, specify the print(s): (x3) | ○ | ○ | ○ | ○ | ○ |
| Social media (Facebook, Instagram, Twitter, etc.). If yes, specify the platform(s): (x4) | ○ | ○ | ○ | ○ | ○ |
| Websites. If yes, specify the website(s): (x5) | ○ | ○ | ○ | ○ | ○ |
| Healthcare professionals and officials (i.e. doctor, nurse, paramedic, etc.). If yes, specify the professional/official title(s): (x6) | ○ | ○ | ○ | ○ | ○ |
| Personal relationships (Friends, family, co-workers, etc.). If yes, specify the relationship(s): (x7) | ○ | ○ | ○ | ○ | ○ |
| Religious leader (Pastor, clergy, minister, etc.). If yes, specify the relationship(s): (x8) | ○ | ○ | ○ | ○ | ○ |
| Governor of your state. (x9) | ○ | ○ | ○ | ○ | ○ |

Page Break

FLU **The following questions ask about your opinion on seasonal flu vaccinations.**

FLU1 Have you been vaccinated against influenza (the flu) in the **last year**?

- Yes (2)
- No (0)
- Don't know (1)

FLU2
Do you plan on getting this season’s influenza vaccine **this fall**?

- Yes, I am planning on receiving the vaccine. (2)
- No, I am not planning on receiving the vaccine. (0)
- Undecided (1)

Page Break

COVID_vir The following questions ask about your knowledge on the COVID-19 virus.

COVID_vir1
Which of the following may protect you from or reduce the risk of transmission of COVID-19? ***Select all that apply.***

- ☐ Taking vitamin and mineral supplements. (1)
- ☐ Treatment with hydroxychloroquine. (2)
- ☐ Treatment with dexamethasone. (3)
- ☐ Drinking alcohol. (4)
- ☐ Adding pepper or eating spicy foods. (5)
- ☐ Spraying a disinfectant on your body. (6)
- ☐ Drinking bleach. (7)
- ☐ Exposing self to sun or high temperatures (greater than 77°F/25°C). (8)
- ☐ Taking a hot bath. (9)
- ☐ Hand dryers. (10)
- ☐ Vaccination against pneumonia. (11)
- ☐ Rinsing your nose with saline. (12)
- ☐ Eating garlic. (13)
- ☐ Antibiotics. (14)
- ☐ Handwashing. (15)
- ☐ Wearing a mask that covers your nose, mouth, and chin. (16)
- ☐ None of the above. (17)

☐ Don't know. (18)

COVID_vir2 Which of the following ways is COVID-19 spread? ***Select <u>all</u> that apply.***

☐ Water or swimming. (1)

☐ Through houseflies. (2)

☐ Through mosquito bites. (3)

☐ Through 5G mobile networks. (4)

☐ Through hot and humid climates. (5)

☐ Between people less than 6 feet of one another. (6)

☐ Through respiratory droplets when an infected person coughs, sneezes, or talks. (7)

☐ By touching a contaminated surface and then touching your eyes, nose, or mouth before washing your hands. (8)

☐ None of the above. (9)

☐ Don't know. (10)

Page Break

COVID_vac **The following questions ask about your knowledge on the COVID-19 vaccines.**

---

COVID_vac1 Vaccines (shots)… *Select <u>all</u> that apply.*

- ☐ Work with your body's natural defenses so your body will be ready to fight the virus. (1)
- ☐ Cause autism. (2)
- ☐ Cause death. (5)
- ☐ Aren't worth the risk. (3)
- ☐ Are unnecessary because infection rates are already low in the United States. (4)
- ☐ Make our body magnetic. (6)
- ☐ None of the above. (7)

---

COVID_vac2 The COVID-19 vaccines…*Select <u>all</u> that apply.*

- ☐ Will prevent you from getting COVID-19 or will help you from getting seriously ill even if you get COVID-19. (9)
- ☐ Will not be effective if you have already had COVID-19. (10)
- ☐ Will give you COVID-19. (11)
- ☐ Are not safe because they were rapidly developed. (12)
- ☐ None of the above. (13)

COVID_vac3 After I get my COVID-19 vaccine, I will…***Select <u>all</u> that apply.***

- ☐ Still be cautious in public.  (2)
- ☐ Become infected and sick.  (3)
- ☐ Be controlled by the government through microchip tracking.  (4)
- ☐ Die from the substances inside the vaccine.  (5)
- ☐ None of the above.  (6)

COVID_vac4 The most common side effects of the COVID-19 vaccines are…
***Select <u>all</u> that apply.***

- ☐ Sore muscles.  (1)
- ☐ Feeling tired.  (5)
- ☐ Mild fever.  (6)
- ☐ None of the above.  (7)

COVID_vac5 Although the COVID-19 vaccines were developed in a faster process than usual, they…*Select all that apply.*

- ☐ Were extensively tested for both safety and efficacy. (1)
- ☐ Have met national government safety standards. (2)
- ☐ Will be carefully monitored to detect any problems or side effects. (3)
- ☐ None of the above. (5)

Page Break

COVID_vacc **The following questions ask about your opinion on the COVID-19 vaccines.**

---

COVID_vac6 If given the opportunity to take a COVID-19 vaccine, how likely is it that you would get the vaccine/shot?

- Definitely will not (1)
- Very unlikely (2)
- Somewhat unlikely (3)
- Somewhat likely (4)
- Very likely (5)
- Definitely will (6)
- I have already received the first dose of a two dose COVID-19 vaccine (i.e. Pfizer-BioNTech, Moderna). (7)
- I have already received both doses of a two dose COVID-19 vaccine (i.e. Pfizer-BioNTech, Moderna). (8)
- I have already received one dose of a one dose COVID-19 vaccine (i.e. Johnson & Johnson's Janssen). (9)

---

COVID_vac7 How concerned are/were you about taking a COVID-19 vaccine?

- Very concerned (4)
- Somewhat concerned (3)
- Slightly concerned (2)
- Not concerned at all (1)

COVID_vac8 Please select ‘**AGREE**,’ ‘**DISAGREE**,’ or ‘**UNSURE’** for the following statements.

| | AGREE (2) | DISAGREE (0) | UNSURE (1) |
|---|---|---|---|
| I am concerned that I will be infected by the COVID-19 virus. (2) | ○ | ○ | ○ |
| I believe that CDC recommendations, such as wearing a mask and social distancing, are effective against a COVID-19 infection. (4) | ○ | ○ | ○ |
| Even if I get infected with COVID-19, I believe that I am healthy enough to fight it off. (5) | ○ | ○ | ○ |
| I am concerned about the mild reactions of a COVID-19 infection. (7) | ○ | ○ | ○ |
| I am concerned about the severe reactions of a COVID-19 infection. (8) | ○ | ○ | ○ |
| I trust that a COVID-19 vaccine will give me immunity against a COVID-19 infection because it is safe and effective against a COVID-19 infection. (1) | ○ | ○ | ○ |
| I believe that it will benefit my body if I take a COVID-19 vaccine. (3) | ○ | ○ | ○ |
| I have been offered a COVID-19 vaccine. (9) | ○ | ○ | ○ |
| I believe that the COVID-19 vaccines have adequate testing and results. (10) | ○ | ○ | ○ |
| The COVID-19 vaccines were developed and tested too quickly. (11) | ○ | ○ | ○ |
| I am concerned about the side effects of a COVID-19 vaccine. (12) | ○ | ○ | ○ |

| | | | |
|---|---|---|---|
| I am concerned about the long-term side effects of a COVID-19 vaccine. (13) | ○ | ○ | ○ |
| It is hard to know whom to trust when it comes to COVID-19 vaccine information. (15) | ○ | ○ | ○ |
| I have previously had a negative experience with other vaccines. (18) | ○ | ○ | ○ |
| I trust the healthcare experts/providers/system. (14) | ○ | ○ | ○ |
| I have personally experienced mistreatment in the medical care system in the past. (16) | ○ | ○ | ○ |
| Historical mistreatment of Black people in the medical care system makes me concerned about the COVID-19 vaccines. (17) | ○ | ○ | ○ |
| My family/friends/co-workers have told me to receive a COVID-19 vaccine. (19) | ○ | ○ | ○ |
| My family and friends do not plan to get a COVID-19 vaccine. (20) | ○ | ○ | ○ |
| My place of work/school/institution has told me to receive a COVID-19 vaccine. (21) | ○ | ○ | ○ |
| A source that I trust (i.e. news, website, etc.) told me to receive a COVID-19 vaccine. (22) | ○ | ○ | ○ |
| I believe it is better for my family, friends, and community if I receive a COVID-19 vaccine. (23) | ○ | ○ | ○ |

| | | | |
|---|---|---|---|
| I do not know where to sign up for a COVID-19 vaccine. (24) | ○ | ○ | ○ |
| It will be difficult to travel to a COVID-19 vaccination site. (25) | ○ | ○ | ○ |
| I do not have health insurance to pay for a COVID-19 vaccine. (26) | ○ | ○ | ○ |
| I am worried about taking time off from my work/schedule to get a COVID-19 vaccine. (27) | ○ | ○ | ○ |
| I am worried about missing work if the side effects of a COVID-19 vaccine make me sick. (28) | ○ | ○ | ○ |
| I can find care for my dependent/child so that I can receive a COVID-19 vaccine. (29) | ○ | ○ | ○ |
| I am hesitant to get a COVID-19 vaccine because of my religious beliefs. (30) | ○ | ○ | ○ |

**End of Block: Pre-survey**

---

**Start of Block: Educational content: Video**

time_video Timing
First Click (1)
Last Click (2)
Page Submit (3)
Click Count (4)

---

VIDEO Watch the video below before continuing with the survey.
You will not be able to proceed with the survey until the duration of the video is complete.

Refreshing the page will restart the timer and delay the survey time.

End of Block: Educational content: Video

---

Start of Block: Educational content: Infographic

time_infographic Timing
First Click  (1)
Last Click  (2)
Page Submit  (3)
Click Count  (4)

INFO Read the infographic below before continuing with the survey.
 You will be given <u>at least 4 minutes</u> to thoroughly read all of the information presented. You will not be able to proceed with the survey until this duration is complete.
Refreshing the page will restart the timer and delay the survey time.

End of Block: Educational content: Infographic

---

Start of Block: Post-survey

COVID_vir The following questions ask about your knowledge on the COVID-19 virus.

COVID_vir1
Which of the following may protect you from or reduce the risk of transmission of COVID-19?
***Select <u>all</u> that apply.***

- ☐ Taking vitamin and mineral supplements. (1)
- ☐ Treatment with hydroxychloroquine. (2)
- ☐ Treatment with dexamethasone. (3)
- ☐ Drinking alcohol. (4)
- ☐ Adding pepper or eating spicy foods. (5)
- ☐ Spraying a disinfectant on your body. (6)
- ☐ Drinking bleach. (7)
- ☐ Exposing self to sun or high temperatures (greater than 77°F/25°C). (8)
- ☐ Taking a hot bath. (9)
- ☐ Hand dryers. (10)
- ☐ Vaccination against pneumonia. (11)
- ☐ Rinsing your nose with saline. (12)
- ☐ Eating garlic. (13)
- ☐ Antibiotics. (14)
- ☐ Handwashing. (15)
- ☐ Wearing a mask that covers your nose, mouth, and chin. (16)
- ☐ None of the above. (17)

☐ Don't know. (18)

COVID_vir2 Which of the following ways is COVID-19 spread? ***Select all that apply.***

☐ Water or swimming. (1)

☐ Through houseflies. (2)

☐ Through mosquito bites. (3)

☐ Through 5G mobile networks. (4)

☐ Through hot and humid climates. (5)

☐ Between people less than 6 feet of one another. (6)

☐ Through respiratory droplets when an infected person coughs, sneezes, or talks. (7)

☐ By touching a contaminated surface and then touching your eyes, nose, or mouth before washing your hands. (8)

☐ None of the above. (9)

☐ Don't know. (10)

Page Break

COVID_vac_post **The following questions ask about your knowledge on the COVID-19 vaccines.**

---

COVID_vac_post1 Vaccines (shots)… *Select all that apply.*

- ☐ Work with your body's natural defenses so your body will be ready to fight the virus. (1)
- ☐ Cause autism. (2)
- ☐ Cause death. (5)
- ☐ Aren't worth the risk. (3)
- ☐ Are unnecessary because infection rates are already low in the United States. (4)
- ☐ Make our body magnetic. (6)
- ☐ None of the above. (7)

---

COVID_vac_post2 The COVID-19 vaccines…*Select all that apply.*

- ☐ Will prevent you from getting COVID-19 or will help you from getting seriously ill even if you get COVID-19. (9)
- ☐ Will not be effective if you have already had COVID-19. (10)
- ☐ Will give you COVID-19. (11)
- ☐ Are not safe because they were rapidly developed. (12)
- ☐ None of the above. (13)

COVID_vac_post3 After I get my COVID-19 vaccine, I will…***Select <u>all</u> that apply.***

- ☐ Still be cautious in public.  (2)
- ☐ Become infected and sick.  (3)
- ☐ Be controlled by the government through microchip tracking.  (4)
- ☐ Die from the substances inside the vaccine.  (5)
- ☐ None of the above.  (6)

COVID_vac_post4 The most common side effects of the COVID-19 vaccines are…
***Select <u>all</u> that apply.***

- ☐ Sore muscles.  (1)
- ☐ Feeling tired.  (5)
- ☐ Mild fever.  (6)
- ☐ None of the above.  (7)

COVID_vac_post5 Although the COVID-19 vaccines were developed in a faster process than usual, they…*Select all that apply.*

- ☐ Were extensively tested for both safety and efficacy. (1)
- ☐ Have met national government safety standards. (2)
- ☐ Will be carefully monitored to detect any problems or side effects. (3)
- ☐ None of the above. (5)

Page Break

COVID_vac_post_b **The following questions ask about your opinion on the COVID-19 vaccines.**

---

X→

COVID_vac_post6 If given the opportunity to take a COVID-19 vaccine, how likely is it that you would get the vaccine/shot?

○ Definitely will not (1)

○ Very unlikely (2)

○ Somewhat unlikely (3)

○ Somewhat likely (4)

○ Very likely (5)

○ Definitely will (6)

○ I have already received the first dose of a two dose COVID-19 vaccine (i.e. Pfizer-BioNTech, Moderna). (7)

○ I have already received both doses of a two dose COVID-19 vaccine (i.e. Pfizer-BioNTech, Moderna). (8)

○ I have already received one dose of a one dose COVID-19 vaccine (i.e. Johnson & Johnson's Janssen). (9)

---

X→

COVID_vac_post7 How concerned are/were you about taking a COVID-19 vaccine?

- Very concerned (4)
- Somewhat concerned (3)
- Slightly concerned (2)
- Not concerned at all (1)

COVID_vac_post8 Please select ‘**AGREE**,’ ‘**DISAGREE**,’ or ‘**UNSURE’** for the following statements.

| | AGREE (2) | DISAGREE (0) | UNSURE (1) |
|---|---|---|---|
| I am concerned that I will be infected by the COVID-19 virus. (2) | ○ | ○ | ○ |
| I believe that CDC recommendations, such as wearing a mask and social distancing, are effective against a COVID-19 infection. (4) | ○ | ○ | ○ |
| Even if I get infected with COVID-19, I believe that I am healthy enough to fight it off. (5) | ○ | ○ | ○ |
| I am concerned about the mild reactions of a COVID-19 infection. (7) | ○ | ○ | ○ |
| I am concerned about the severe reactions of a COVID-19 infection. (8) | ○ | ○ | ○ |
| I trust that a COVID-19 vaccine will give me immunity against a COVID-19 infection because it is safe and effective against a COVID-19 infection. (1) | ○ | ○ | ○ |
| I believe that it will benefit my body if I take a COVID-19 vaccine. (3) | ○ | ○ | ○ |
| I have been offered a COVID-19 vaccine. (9) | ○ | ○ | ○ |
| I believe that the COVID-19 vaccines have adequate testing and results. (10) | ○ | ○ | ○ |
| The COVID-19 vaccines were developed and tested too quickly. (11) | ○ | ○ | ○ |
| I am concerned about the side effects of a COVID-19 vaccine. (12) | ○ | ○ | ○ |

| | | | |
|---|---|---|---|
| I am concerned about the long-term side effects of a COVID-19 vaccine. (13) | o | o | o |
| It is hard to know whom to trust when it comes to COVID-19 vaccine information. (15) | o | o | o |
| I have previously had a negative experience with other vaccines. (18) | o | o | o |
| I trust the healthcare experts/providers/system. (14) | o | o | o |
| I have personally experienced mistreatment in the medical care system in the past. (16) | o | o | o |
| Historical mistreatment of Black people in the medical care system makes me concerned about the COVID-19 vaccines. (17) | o | o | o |
| My family/friends/co-workers have told me to receive a COVID-19 vaccine. (19) | o | o | o |
| My family and friends do not plan to get a COVID-19 vaccine. (20) | o | o | o |
| My place of work/school/institution has told me to receive a COVID-19 vaccine. (21) | o | o | o |
| A source that I trust (i.e. news, website, etc.) told me to receive a COVID-19 vaccine. (22) | o | o | o |
| I believe it is better for my family, friends, and community if I receive a COVID-19 vaccine. (23) | o | o | o |

| | | | |
|---|---|---|---|
| I do not know where to sign up for a COVID-19 vaccine. (24) | ○ | ○ | ○ |
| It will be difficult to travel to a COVID-19 vaccination site. (25) | ○ | ○ | ○ |
| I do not have health insurance to pay for a COVID-19 vaccine. (26) | ○ | ○ | ○ |
| I am worried about taking time off from my work/schedule to get a COVID-19 vaccine. (27) | ○ | ○ | ○ |
| I am worried about missing work if the side effects of a COVID-19 vaccine make me sick. (28) | ○ | ○ | ○ |
| I can find care for my dependent/child so that I can receive a COVID-19 vaccine. (29) | ○ | ○ | ○ |
| I am hesitant to get a COVID-19 vaccine because of my religious beliefs. (30) | ○ | ○ | ○ |

**End of Block: Post-survey**
