# Supplementary material for: Evaluating Two Educational Interventions for Enhancing COVID-19 Knowledge and Attitudes in a Sample American Indian/Alaska Native Population
Source: Vaccines (Basel). 2024 Jul 17;12(7):787. doi: 10.3390/vaccines12070787 (PMC11281502; doi:10.3390/vaccines12070787)
Supplement: Supplementary file 1 [file vaccines-12-00787-s001.zip › Supplementary Tables.pdf]

Supplementary Tables

Table S1. Mean difference ($\Delta\bar{x}$) of significant virus and vaccine knowledge topics based on gender, political affiliation, and employment status.

	**Gender**				**Political affiliation**						**Employment status**			
**Knowledge topics**	**Female ($\Delta\bar{x}$)**	**Male ($\Delta\bar{x}$)**	**t**	**p-value**	**Republican ($\Delta\bar{x}$)**	**Democrat ($\Delta\bar{x}$)**	**Independent ($\Delta\bar{x}$)**	**Something else [illegible]**	**F**	**p-value**	**Unaffected ($\Delta\bar{x}$)**	**Affected ($\Delta\bar{x}$)**	**t**	**p-value**
**Measures to protect against COVID-19 transmission**	0.27	0.29	-0.14	0.888	0.23	0.20	0.29	0.37	0.73	0.536	0.31	0.26	0.82	0.599
**Vaccine effects**	0.19	0.03	1.34	0.183	0.05	0.11	0.12	0.20	0.32	0.813	0.05	0.25	0.86	**0.075**
**COVID-19 vaccine efficacy**	0.22	0.10	1.08	0.283	0.53	0.06	0.12	0.18	3.05	**0.029**	0.08	0.30	0.91	**0.043**
**Post-vaccine behavior**	0.21	-0.06	2.48	**0.014**	0.03	0.10	0.31	0.06	1.51	0.213	0.08	0.19	0.90	0.353
**COVID-19 vaccine development**	0.20	0.17	0.21	0.834	0.03	0.00	0.25	0.40	3.50	**0.016**	0.18	0.21	0.94	0.786

Table S2A. Non-significant mean differences ($\Delta\bar{x}$) of virus and vaccine knowledge topics based on age, residence, and essential worker status of the participant. The Pearson correlation coefficient (*r*) of the number of CDC precautions that the participant follows versus correctness of COVID-19 knowledge topics.

	Age								Residence					Essential worker					CDC	
**Knowledge topics**	**18 – 24 years old ($\Delta\bar{x}$)**	**25 – 34 years old ($\Delta\bar{x}$)**	**35 – 44 years old ($\Delta\bar{x}$)**	**45 – 54 years old ($\Delta\bar{x}$)**	**55 – 64 years old ($\Delta\bar{x}$)**	**65+ years old ($\Delta\bar{x}$)**	**F**	**p-value**	**Nonmetropolitan ($\Delta\bar{x}$)**	**Micropolitan ($\Delta\bar{x}$)**	**Metropolitan ($\Delta\bar{x}$)**	**F**	**p-value**	**Self and/or household ($\Delta\bar{x}$)**	**Household only ($\Delta\bar{x}$)**	**Neither ($\Delta\bar{x}$)**	**F**	**p-value**	**precautions (*r*)**	**p-value**
**Measures to protect against COVID-19 transmission**	0.44	0.48	0.21	0.18	0.20	0.07	1.94	0.089	0.44	0.48	0.21	0.18	0.936	0.1628	0.3396	0.3358	1.512	0.222	0.02	0.750
**Vaccine effects**	0.08	0.27	0.21	0.11	-0.05	0.14	0.78	0.564	0.08	0.27	0.21	0.11	0.387	0.186	0.1698	0.097	0.315	0.73	0.05	0.420
**COVID-19 vaccine efficacy**	0.21	0.22	0.23	0.20	-0.02	0.32	0.76	0.578	0.21	0.22	0.23	0.20	0.219	0.1744	0.3019	0.1493	0.661	0.517	0.06	0.340
**Post-vaccine behavior**	0.08	0.10	0.26	0.13	0.14	-0.07	0.67	0.646	0.08	0.10	0.26	0.13	0.453	0.2907	0.1321	0.0149	2.961	**0.053**	-0.06	0.330
**COVID-19 vaccine development**	0.15	0.25	0.33	0.11	0.09	0.14	0.56	0.735	0.15	0.25	0.33	0.11	0.123	0.1395	0.1509	0.2463	0.454	0.636	-0.07	0.220

Table S2B. Non-significant mean differences (Δx̄) of virus and vaccine knowledge topics based on if the participant had ever received a COVID-19 test, had a positive COVID-19 test, were or knew someone who was hospitalized due to COVID-19, and number of co-morbidities of the participant themselves and/or someone in their household that would put them at risk for a severe COVID-19 infection.

	**COVID-19 tested**				**Positive COVID-19 test**				**COVID-19 hospitalization (self or other)**				**Risk co-morbidities (self)**					**Risk co-morbidities (other)**				
**Knowledge topics**	**No/Don't know (Δx̄)**	**Yes (Δx̄)**	**t**	**p-value**	**No/Don't know (Δx̄)**	**Yes (Δx̄)**	**t**	**p-value**	**No/Don't know (Δx̄)**	**Yes (Δx̄)**	**t**	**p-value**	**None (Δx̄)**	**One (Δx̄)**	**Two or more (Δx̄)**	**F**	**p-value**	**None (Δx̄)**	**One (Δx̄)**	**Two or more (Δx̄)**	**F**	**p-value**
**Measures to protect against COVID-19 transmission**	0.24	0.29	-0.378	0.706	0.30	0.28	0.10	0.920	0.37	0.22	1.526	0.128	0.30	0.26	0.30	0.10	0.906	0.29	0.28	0.28	0.01	0.991
**Vaccine effects**	0.15	0.14	0.056	0.955	0.30	0.12	0.99	0.322	0.16	0.13	0.281	0.779	0.20	0.17	0.07	0.49	0.612	0.15	0.15	0.11	0.07	0.929
**COVID-19 vaccine efficacy**	0.24	0.18	0.365	0.715	0.41	0.16	1.47	0.144	0.16	0.21	-0.516	0.606	0.20	0.25	0.09	1.03	0.358	0.15	0.26	0.12	0.82	0.440
**Post-vaccine behavior**	0.38	0.09	1.954	0.052	0.22	0.11	0.65	0.519	0.10	0.14	-0.344	0.731	0.14	0.19	0.02	1.09	0.338	0.15	0.15	0.08	0.21	0.813
**COVID-19 vaccine development**	0.21	0.19	0.082	0.935	-0.04	0.22	-1.43	0.155	0.17	0.21	-0.32	0.749	0.09	0.26	0.17	0.78	0.460	0.09	0.20	0.26	0.66	0.519

Table S3. Non-significant mean differences ($\Delta\bar{x}$) of COVID-19 virus and vaccine attitude topics that did not show significant changes following the educational interventions.

Attitude topics	Mean difference ($\Delta\bar{x}$)	p-value
**Concerns about COVID-19 infection risk**	-0.07	0.167
**Trust in CDC recommendations for COVID-19 prevention**	0.02	0.706
**Confidence in personal ability to fight off COVID-19**	0.01	0.772
**Concerns about mild reactions to COVID-19 infection**	0.05	0.436
**Concerns about severe reactions to COVID-19 infection**	-0.02	0.78
**Availability of COVID-19 vaccines**	-0.04	0.152
**Influence of past negative vaccine experiences**	0.03	0.415
**Trust in healthcare experts/providers/system**	0.03	0.410
**Past mistreatment experiences in the medical care system**	-0.04	0.367
**Historical mistreatment concerns affecting vaccine trust**	-0.05	0.187
**Influence of family, friends, and co-workers on vaccination**	0.07	0.156
**Resistance to COVID-19 vaccination among peers**	0.05	0.227
**Influence of workplace/school/institutional policies on vaccination**	0.06	0.180
**Perceived benefits of COVID-19 vaccination for community health**	0.04	0.272
**Uncertainty about COVID-19 vaccine sign-up procedures**	0.04	0.318
**Challenges in accessing vaccination sites**	-0.04	0.275
**Affordability of COVID-19 vaccination without health insurance**	-0.04	0.313
**Concerns about taking time off work/schedule for vaccination**	0.03	0.434
**Arranging care for dependents/children during vaccination**	-0.02	0.786
**Religious beliefs impacting COVID-19 vaccine acceptance**	0.00	1.00

Table S4A. Mean difference (Δx̄) of significant virus and vaccine attitude topics based on gender, residence, and if the participant had ever received a COVID-19 test.

	Gender				Residence					COVID-19 tested			
**Attitude topics**	**Female (Δx̄)**	**Male (Δx̄)**	**t**	**p-value**	**Nonmetropolitan (Δx̄)**	**Micropolitan (Δx̄)**	**Metropolitan (Δx̄)**	**F**	**p-value**	**No/Don't know (Δx̄)**	**Yes (x̄)**	**t**	**p-value**
**Trust in COVID-19 vaccine effectiveness and safety**	0.19	0.10	0.95	0.342	0.16	0.18	0.03	0.56	0.571	0.35	0.14	1.46	0.145
**Personal belief in the benefits of COVID-19 vaccination**	0.09	0.07	0.21	0.838	0.11	0.07	0.12	0.12	0.884	0.21	0.07	1.27	0.204
**Confidence in COVID-19 vaccine testing and results**	0.10	0.08	0.17	0.865	0.06	0.09	0.12	0.06	0.941	-0.06	0.12	-1.57	0.118
**Perception of rapid COVID-19 vaccine development**	-0.11	-0.07	-0.46	0.646	-0.11	-0.09	-0.19	0.29	0.748	-0.25	-0.08	-1.27	0.205
**Concerns about COVID-19 vaccine side effects**	-0.05	-0.30	2.31	**0.021**	-0.32	-0.06	-0.47	4.37	**0.014**	-0.25	-0.11	-0.89	0.377
**Concerns about long-term effects of COVID-19 vaccines**	-0.09	-0.32	2.32	**0.021**	-0.28	-0.11	-0.41	2.70	0.069	-0.42	-0.12	-2.09	**0.038**
**Influence of trusted sources on vaccination**	0.15	0.11	0.35	0.724	0.37	0.11	0.16	1.02	0.361	0.13	0.13	-0.03	0.973
**Concerns about missing work due to vaccine side effects**	-0.09	-0.07	-0.20	0.839	0.05	-0.11	-0.03	0.55	0.576	-0.06	-0.09	0.21	0.837

Table S4B. Mean difference ($\Delta\bar{x}$) of significant virus and vaccine attitude topics based on number of co-morbidities of the participant themselves that would put them at risk for a severe COVID-19 infection. The Pearson correlation coefficient (*r*) of the number of CDC precautions that the participant follows versus COVID-19 attitude topics.

	Risk co-morbidities (self)					CDC	
**Attitude topics**	**None ($\Delta\bar{x}$)**	**One ($\Delta\bar{x}$)**	**Two or more ($\Delta\bar{x}$)**	**F**	**p-value**	**Precautions (*r*)**	**p-value**
**Trust in COVID-19 vaccine effectiveness and safety**	0.06	0.17	0.22	0.75	0.473	0.05	0.42
**Personal belief in the benefits of COVID-19 vaccination**	0.23	0.09	-0.01	3.25	**0.04**	-0.02	0.78
**Confidence in COVID-19 vaccine testing and results**	0.08	0.10	0.11	0.04	0.963	-0.05	0.45
**Perception of rapid COVID-19 vaccine development**	-0.10	0.02	-0.28	4.70	**0.01**	-0.07	0.25
**Concerns about COVID-19 vaccine side effects**	-0.25	-0.01	-0.24	2.69	0.07	0.11	0.09
**Concerns about long-term effects of COVID-19 vaccines**	-0.10	-0.03	-0.39	6.17	**0.002**	0.01	0.88
**Influence of trusted sources on vaccination**	0.10	0.16	0.12	0.15	0.864	-0.02	0.72
**Concerns about missing work due to vaccine side effects**	-0.10	-0.04	-0.14	0.53	0.591	0.15	**0.02**

Table S5A. Non-significant mean differences ($\Delta\bar{x}$) of virus and vaccine attitude topics based on types of intervention assigned, age, political affiliation, and employment status.

	Educational intervention				Age								Political affiliation						Employment status			
**Attitude topics**	**Video ($\Delta\bar{x}$)**	**Infographic ($\Delta\bar{x}$)**	**t**	**p-value**	**18 – 24 years old ($\Delta\bar{x}$)**	**25 – 34 years old ($\Delta\bar{x}$)**	**35 – 44 years old ($\Delta\bar{x}$)**	**45 – 54 years old ($\Delta\bar{x}$)**	**55 – 64 years old ($\Delta\bar{x}$)**	**65+ years old ($\Delta\bar{x}$)**	**F**	**p-value**	**Republican ($\Delta\bar{x}$)**	**Democrat ($\Delta\bar{x}$)**	**Independent ($\Delta\bar{x}$)**	**Something else ($\Delta\bar{x}$)**	**F**	**p-value**	**Unaffected ($\Delta\bar{x}$)**	**Affected ($\Delta\bar{x}$)**	**t**	**p-value**
**Trust in COVID-19 vaccine effectiveness and safety**	0.14	0.19	-0.54	0.591	0.10	0.21	0.19	0.19	0.07	0.24	0.27	0.930	0.16	0.21	-0.02	0.28	1.79	0.151	0.17	0.16	0.10	0.920
**Personal belief in the benefits of COVID-19 vaccination**	0.13	0.04	1.32	0.19	0.05	0.18	0.11	0.03	0.17	-0.15	1.67	0.142	0.13	0.01	0.13	0.10	0.66	0.577	0.04	0.15	-1.45	0.148
**Confidence in COVID-19 vaccine testing and results**	0.11	0.08	0.44	0.659	0.03	0.10	0.19	0.20	-0.02	0.04	0.90	0.480	0.08	0.08	0.02	0.17	0.80	0.495	0.13	0.06	0.75	0.452
**Perception of rapid COVID-19 vaccine development**	-0.12	-0.09	-0.37	0.711	-0.05	-0.15	0.06	-0.20	-0.12	-0.22	0.97	0.436	-0.03	-0.09	-0.16	-0.14	0.34	0.795	-0.06	-0.13	0.78	0.439
**Concerns about COVID-19 vaccine side effects**	-0.11	-0.15	0.34	0.736	-0.05	-0.05	-0.07	-0.13	-0.20	-0.44	1.10	0.363	-0.03	-0.17	-0.16	-0.11	0.32	0.812	-0.01	-0.21	1.86	**0.064**
**Concerns about long-term effects of COVID-19 vaccines**	-0.10	-0.21	1.22	0.224	-0.05	-0.03	-0.07	-0.38	-0.31	-0.23	1.70	0.136	-0.03	-0.12	-0.32	-0.14	1.47	0.224	-0.14	-0.16	0.15	0.884
**Influence of trusted sources on vaccination**	0.17	0.10	0.78	0.437	0.33	0.02	0.21	0.00	0.17	0.08	1.20	0.312	0.08	0.14	0.10	0.19	0.26	0.854	0.15	0.14	0.06	0.951
**Concerns about missing work due to vaccine side effects**	-0.07	-0.10	0.26	0.793	0.08	0.02	-0.23	-0.08	-0.23	-0.08	1.44	0.209	-0.08	-0.11	-0.15	-0.03	0.35	0.787	0.01	-0.16	1.75	0.082

Table S5B. Non-significant mean differences (Δx̄) of virus and vaccine attitude topics based on essential worker status, if the participant had had a positive COVID-test, were or knew someone who was hospitalized due to COVID, and number of co-morbidities of the someone in their household that would put them at risk for a severe COVID-19 infection.

	Essential worker					Positive COVID-19 test				COVID-19 hospitalization (self or other)				Risk co-morbidities (other)				
**Attitude topics**	**Self and/or household (Δx̄)**	**Household only (Δx̄)**	**Neither**	**F**	**p-value**	**No/Don't know (Δx̄)**	**Yes (Δx̄)**	**t**	**p-value**	**No/Don't know (Δx̄)**	**Yes (Δx̄)**	**t**	**p-value**	**None (Δx̄)**	**One (Δx̄)**	**Two or more (Δx̄)**	**F**	**p-value**
**Trust in COVID-19 vaccine effectiveness and safety**	0.14	0.22	0.16	0.16	0.851	0.40	0.14	1.61	0.11	0.18	0.15	0.32	0.748	0.03	0.21	0.20	1.15	0.318
**Personal belief in the benefits of COVID-19 vaccination**	0.12	0.12	0.05	0.60	0.55	0.28	0.06	1.89	0.061	0.14	0.05	1.36	0.176	0.15	0.09	0.04	0.72	0.488
**Confidence in COVID-19 vaccine testing and results**	0.00	0.10	0.16	1.71	0.183	-0.04	0.11	-1.16	0.246	0.12	0.08	0.54	0.588	0.13	0.05	0.13	0.55	0.576
**Perception of rapid COVID-19 vaccine development**	-0.11	-0.14	-0.09	0.09	0.911	-0.04	-0.11	0.46	0.644	-0.15	-0.07	-0.88	0.379	-0.05	-0.06	-0.19	1.17	0.312
**Concerns about COVID-19 vaccine side effects**	-0.19	-0.06	-0.12	0.45	0.637	-0.42	-0.10	-1.81	0.071	-0.16	-0.11	-0.44	0.657	-0.17	-0.08	-0.16	0.28	0.753
**Concerns about long-term effects of COVID-19 vaccines**	-0.22	0.02	-0.19	1.89	0.154	-0.42	-0.13	-1.79	0.075	-0.15	-0.17	0.22	0.825	-0.13	-0.17	-0.17	0.05	0.948
**Influence of trusted sources on vaccination**	0.20	0.20	0.06	1.02	0.363	0.09	0.14	-0.31	0.76	0.12	0.14	-0.21	0.833	0.07	0.17	0.13	0.37	0.691
**Concerns about missing work due to vaccine side effects**	-0.11	-0.10	-0.06	0.13	0.879	-0.17	-0.08	-0.58	0.56	-0.07	-0.10	0.39	0.695	-0.12	-0.04	-0.12	0.38	0.682

vaccines

MDPI

Table S6. Non-significant mean differences ($\Delta\bar{x}$) of the likelihood of receiving a COVID-19 vaccine and concerns for receiving the COVID-19 vaccine.

Likelihood[1] and concern[2]	Mean difference ($\Delta\bar{x}$)	Standard deviation	t	p-value
**Likelihood of receiving COVID-19 vaccine (*All participants*[3], *N=273*)**	-0.04	1.17	-0.63	0.531
**Likelihood of receiving COVID-19 vaccine (*Unvaccinated participants only*[4], *N=96*)**	0.07	0.94	0.76	0.451
**Concern for receiving COVID-19 vaccine (*N=264*)**	-0.07	0.96	-1.22	0.225

[1] The "Likelihood of receiving the COVID-19 vaccine" scale ranged from "Definitely will not" = 1, "Very unlikely" = 2, "Somewhat unlikely" = 3, "Somewhat likely" = 4, "Very likely" = 5, or "Definitely will" = 6.

[2] The "Concern for receiving the COVID-19 vaccine" scale ranged from "Not concerned at all" = 1, "Slightly concerned" = 2, "Somewhat concerned" = 3, and "Very concerned" = 4.

[3] *"All participants" include individuals who selected "Definitely will not," "Very unlikely," "Somewhat unlikely," "Somewhat likely," "Very likely," "Definitely will," and "Already vaccinated" to the question, "If given the opportunity to take a COVID-19 vaccine, how likely is it that you would get the vaccine/shot?"

[4] "Unvaccinated participants only" includes individuals who selected "Definitely will not," "Very unlikely," "Somewhat unlikely," "Somewhat likely," "Very likely," or "Definitely will" to the question, "If given the opportunity to take a COVID-19 vaccine, how likely is it that you would get the vaccine/shot?"

Table S7. Non-significant mean differences (Δx̄) of virus and vaccine knowledge and attitude topics, as well as mean difference (Δx̄) in likelihood of receiving a COVID-19 vaccine and concerns for COVID-19 items based on type of educational interventions.

	**Educational Intervention**			
	**Video (Δx̄)**	**Infographic (Δx̄)**	**t**	**p-value**
**Knowledge topics**				
**Measures to protect against COVID-19 transmission**	0.29	0.28	0.08	0.939
**Vaccine effects**	0.12	0.16	-0.35	0.726
**COVID-19 vaccine efficacy**	0.18	0.19	-0.12	0.902
**Post-vaccine behavior**	0.11	0.14	-0.38	0.708
**COVID-19 vaccine development**	0.28	0.11	1.53	0.128
**Attitude topics**				
**Trust in COVID-19 vaccine effectiveness and safety**	0.14	0.19	-0.54	0.591
**Personal belief in the benefits of COVID-19 vaccination**	0.13	0.04	1.32	0.19
**Confidence in COVID-19 vaccine testing and results**	0.11	0.08	0.44	0.659
**Perception of rapid COVID-19 vaccine development**	-0.12	-0.09	-0.37	0.711
**Concerns about COVID-19 vaccine side effects**	-0.11	-0.15	0.34	0.736
**Concerns about long-term effects of COVID-19 vaccines**	-0.1	-0.21	1.22	0.224
**Influence of trusted sources on vaccination**	0.17	0.1	0.78	0.437
**Concerns about missing work due to vaccine side effects**	-0.07	-0.1	0.26	0.793
**Likelihood and Concern**				
**Likelihood of getting COVID-19 vaccine**	-0.03	-0.06	0.19	0.85
**Concern for COVID-19 vaccine**	0.05	-0.19	2.11	**0.036**
