# Supplementary figures and images for: Evaluating Two Educational Interventions for Enhancing COVID-19 Knowledge and Attitudes in a Sample American Indian/Alaska Native Population
Source: Vaccines (Basel). 2024 Jul 17;12(7):787. doi: 10.3390/vaccines12070787 (PMC11281502; doi:10.3390/vaccines12070787)

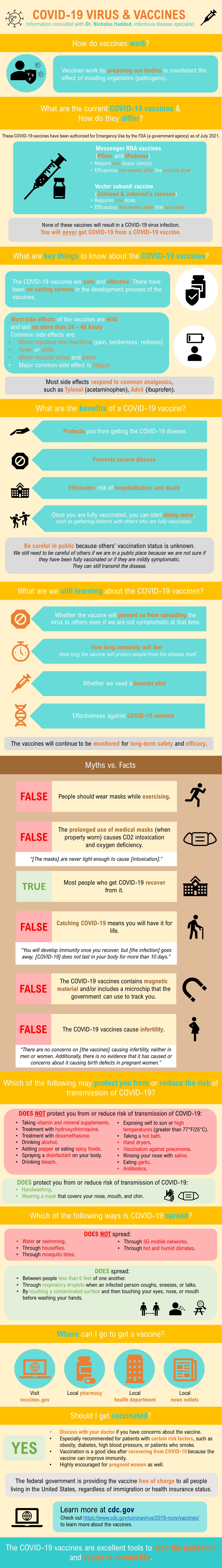

Supplement: Supplementary file 1 [file vaccines-12-00787-s001.zip › Supplementary Material S2.jpg]
